# Global estimates of rotavirus vaccine efficacy and effectiveness: a rapid review and meta-regression analysis

**DOI:** 10.1016/j.eclinm.2025.103122

**Published:** 2025-03-04

**Authors:** Ottavia Prunas, Ernest O. Asare, Elizabeth Sajewski, Yueqi Li, Zeaan Pithawala, Daniel M. Weinberger, Joshua L. Warren, George E. Armah, Nigel A. Cunliffe, Miren Iturriza-Gómara, Benjamin A. Lopman, Virginia E. Pitzer

**Affiliations:** a Swiss Tropical and Public Health Institute, Basel, Switzerland; b University of Basel, Basel, Switzerland; c Department of Epidemiology of Microbial Disease, Yale School of Public Health, Yale University, New Haven, CT, USA; d Public Health Modeling Unit, Yale School of Public Health, Yale University, New Haven, CT, USA; e Department of Biostatistics, Yale School of Public Health, Yale University, New Haven, CT, USA; f Noguchi Memorial Institute for Medical Research, University of Ghana, Accra, Ghana; g Department of Clinical Infection, Microbiology and Immunology, Institute of Infection, Veterinary and Ecological Sciences, University of Liverpool, Liverpool, UK; h Department of Epidemiology, Rollins School of Public Health, Emory University, Atlanta, GA, USA

**Keywords:** Rotavirus-associated gastroenteritis, Immunization, Vaccine effectiveness, Prediction, Model

## Abstract

**Background:**

Rotavirus is the leading cause of diarrhoea worldwide, particularly affecting young children. While national rotavirus immunization programs have reduced rotavirus morbidity and mortality, vaccine performance varies considerably between high-income and low-income settings.

**Methods:**

We updated a previous systematic review of studies reporting rotavirus vaccine efficacy and vaccine effectiveness against severe rotavirus-associated gastroenteritis (RVGE) by performing a rapid review from July 1, 2020 through October 16, 2024. We included randomized controlled trials reporting vaccine efficacy against severe RVGE and case-control and cohort studies reporting vaccine effectiveness against hospitalization with RVGE in children <5 years old for current internationally licensed vaccines. We developed a meta-regression model for vaccine efficacy and effectiveness using widely available country-specific predictors of rotavirus vaccine performance and simultaneously estimated the relationship between vaccine efficacy and effectiveness. We used the model to predict vaccine efficacy and effectiveness for all countries and assessed its predictive accuracy using a modified leave-one-country-out validation approach.

**Findings:**

Predicted vaccine efficacy ranged from 69.6% to 94.3% across countries in the Americas, European, and Western Pacific Regions, with a decreased efficacy ranging from 18.6% to 85.3% in the African, South-East Asian, and Eastern Mediterranean regions. Estimates of vaccine effectiveness were generally lower than vaccine efficacy when efficacy was greater than 60%, but effectiveness was predicted to be higher when vaccine efficacy was low. A strong correlation (*r* = 0.63) was found between the observed and predicted vaccine efficacy and effectiveness, with 98.2% of observed efficacy and effectiveness estimates falling within the 95% prediction intervals.

**Interpretation:**

Our approach enhances the understanding of global variation in rotavirus vaccine performance and can be used to inform predictions of the potential impact of rotavirus vaccines for countries that have yet to introduce them. Higher-quality data on predictor variables and broader regional representation in vaccine trials are required for more robust vaccine performance estimates.

**Funding:**

10.13039/100000002National Institutes of Health/10.13039/100000060National Institute of Allergy and Infectious Diseases (R01AI112970) and the 10.13039/100000865Bill & Melinda Gates Foundation (INV-17940).

**Research in context**

**Evidence before this study**

Multiple systematic reviews have highlighted variability in rotavirus vaccine performance across different settings, with higher efficacy and effectiveness observed in high-income and low-child-mortality countries. We conducted a rapid review, extending a previous systematic review to October 16, 2024. We searched four platforms (PubMed, EMBASE, Cochrane and Web of Science) using the search terms “rotavirus” and “vaccin∗” to identify estimates of vaccine efficacy and effectiveness from randomized controlled trials, cohort studies, and case-control studies for the four currently licensed rotavirus vaccines (RotaTeq, Rotarix, Rotavac and Rotasiil). Previous meta-analyses have reported estimates of rotavirus vaccine efficacy and (direct) effectiveness stratified by either country income level or child mortality percentiles. We also identified several modelling studies, including one that used a meta-regression approach to estimate rotavirus vaccine efficacy by duration of follow-up; however, this study assumed vaccine efficacy was the same within child mortality strata. These estimates of vaccine efficacy were then used to predict the impact of rotavirus vaccination (i.e., overall effectiveness) in different countries, accounting for variation in vaccine coverage, but again vaccine efficacy was assumed to be identical for countries within the same child mortality strata.

**Added value of this study**

This study contributes novel insights by providing the first country-specific global estimates of rotavirus vaccine efficacy and direct effectiveness. We developed a new meta-regression framework, which accounts for multiple country-specific predictors of rotavirus vaccine efficacy rather than only country income level or child mortality strata. We also estimated the relationship between vaccine efficacy and real-world estimates of vaccine effectiveness. This analytical approach serves as a valuable tool for guiding decision-making in countries yet to implement a rotavirus vaccine program and for the sustained implementation of vaccine programs in countries that have already introduced the vaccine.

**Implications of all the available evidence**

Despite uncertainties in the predictions, our findings underscore the potential benefits of rotavirus vaccination. Our results align with the World Health Organization recommendations advocating for universal introduction of rotavirus vaccine into routine infant immunization programs. Rotavirus vaccine performance varied by geographical region, emphasizing the need for improved regional representation in future vaccine clinical trials to ensure reliable efficacy estimates.

## Introduction

Rotavirus vaccination has proven to be an effective intervention strategy against severe rotavirus-associated gastroenteritis (RVGE), and the World Health Organization (WHO) has recommended the introduction of rotavirus vaccines into national immunization programs. Currently, four live oral rotavirus vaccines (RotaTeq, Rotarix, Rotavac, and Rotasiil) are available and prequalified by the WHO. There has been a substantial reduction in severe RVGE morbidity and hospitalizations globally following the introduction of rotavirus vaccines.^1^, ^2^, ^3^ However, there has also been considerable variability in the observed vaccine effectiveness and impact, with lower vaccine effectiveness and heterogeneous impact observed in low- and middle-income countries (LMICs).^4^, ^5^, ^6^, ^7^, ^8^ This disparity is likely explained by a variety of factors, such as age at first infection, co-infections, malnutrition, interference resulting from transplacental maternal antibodies, and co-administration with other vaccines.^1^^,^^9^, ^10^, ^11^, ^12^, ^13^, ^14^ Thus, more efforts are needed to identify strategies to improve vaccine effectiveness and impact across LMICs.

As of December 2023, rotavirus vaccines have been introduced in 123 countries, with an estimated global coverage of 55%.^15^ Despite the significant progress in the introduction of rotavirus vaccines, some high-mortality-burden countries, such as Chad, the Central African Republic, Somalia, and South Sudan, have yet to introduce the vaccine into their national immunization programs.^15^ More realistic country-specific estimates of the potential health benefits of rotavirus vaccines may help to better inform decision-makers in these high-burden countries. Moreover, countries that have already implemented the vaccine can use predictions of its effectiveness and impact to support sustained implementation.

Given the large variability in rotavirus vaccine efficacy and effectiveness and paucity of data from most countries, meta-regression models can be employed to generate more robust, country-specific estimates of vaccine performance. Meta-regression models have proven successful in burden estimation for diseases like typhoid fever,^16^ where incidence data are typically scarce in LMICs, and in analysing diarrhoea-related deaths caused by rotavirus and different diarrhoeal pathogens.^17^, ^18^, ^19^ Here, we developed a meta-regression framework accounting for potential drivers of rotavirus incidence and vaccine performance to predict country-specific rotavirus vaccine efficacy and effectiveness for all countries. We evaluated the predictive accuracy of our model by performing a modified leave-one-country-out and out-of-sample validations.

## Methods

### Data section

#### Search strategy and inclusion criteria

We updated previous systematic reviews of rotavirus vaccine efficacy, defined as vaccine performance under the idealized conditions of a randomized controlled trial (RCT), and vaccine effectiveness, defined as the performance under real-world conditions, by applying similar search criteria and searching articles published from July 1, 2020 through October 16, 2024.^20^ We included RCTs for rotavirus vaccine efficacy and case-control or cohort designs for vaccine effectiveness. We extracted data on case counts, person-time of follow-up, and details of study design, including study years, study country, and participants enrolled (by country in the case of multi-country studies). We also extracted the outcome information, comprised of country-specific estimates of vaccine efficacy and effectiveness against severe RVGE (Vesikari score ≥11) and rotavirus-associated hospitalization, along with their corresponding standard errors (see Supplementary Table S1 in Supplementary Appendix 1 for the inclusion and exclusion criteria). The full list of study references and the characteristics of the included studies are provided in Supplementary Appendix 2. We assessed the quality of the included studies using the Cochrane Risk of Bias tool and the Newcastle-Ottawa Scale.^21^^,^^22^

We obtained data on widely available potential predictors of rotavirus vaccine efficacy and effectiveness from publicly available databases (Institute for Health Metrics and Evaluation, World Bank Open Data, and WHO). Potential predictors were selected based on their relevance to rotavirus transmission and vaccine performance, and widespread availability, including indicators of environmental characteristics and socioeconomic development level. Specifically, we identified ten variables: diarrhoea prevalence among children under five years of age; population density (log-transformed); mortality rate under five years of age; oral polio vaccination (OPV) coverage; gross domestic product (GDP) per capita (log-transformed); population living in extreme poverty; population using at least basic sanitation (use of improved facilities which are not shared with other households); population using at least basic drinking water (drinking water from an improved source, provided collection time is not more than 30 min for a roundtrip) and antibiotic usage^23^ (see Supplementary Appendix 2). We also controlled for the duration of study follow-up (in years) for RCTs and cohort studies and age groups included in the analysis for case-control studies. To account for geographical disparities, studies were categorized by WHO region (African Region [AFR], Region of the Americas [AMR], South-East Asian Region [SEAR], European Region [EUR], Eastern Mediterranean Region [EMR], and Western Pacific Region [WPR]).

### Model framework

We developed a hierarchical Bayesian meta-regression framework to jointly model vaccine efficacy and effectiveness and ultimately allow us to predict both outcomes in locations without such estimates. Working in the hierarchical Bayesian setting also ensures that our predictions correctly characterize multiple sources of uncertainty, resulting in robust point estimates and credible intervals. At the first level of the model, we adopted a traditional meta-analysis approach, where we assumed the log-transformed relative risks and odds ratios (i.e., one minus the vaccine efficacy and effectiveness estimates) were unbiased representations of the true but unobserved outcomes, with uncertainty described by the corresponding standard errors. We considered outcome measures for all four available rotavirus vaccines together, since there are limited studies for Rotavac and Rotasiil and no evidence to suggest that efficacy and effectiveness differs across the vaccines. Because the study-specific estimates were often built on populations residing in multiple countries where efficacy and/or effectiveness may vary, as a second level, we modelled the mean of the distribution of the true study-specific values as a weighted average of country-specific values. The weights were defined based on the proportion of the total population from a specific study residing in each respective country. Next, we incorporated a third level into our model to describe variability in the latent country-specific efficacy values. These effects were modelled as a function of the country-level predictors. Values for country-level predictors were selected for the corresponding study year. A random effect for WHO region was also included to account for correlation between countries within the same region. Finally, given that vaccine efficacy and effectiveness are closely linked, we modelled the country-specific log-transformed odds ratios as a function of the corresponding log-transformed relative risk value, assuming a linear relationship between the two measures. All stages of the framework were fit jointly to allow for the estimation of regression coefficients and imputation of missing efficacy or effectiveness estimates from different countries. Further details are presented in Supplementary Appendix 1 and Fig. S1.

We employed a Bayesian framework for model fitting and used Markov chain Monte Carlo methods via the rjags package^24^ to sample from the joint posterior distribution of all model parameters. Weakly informative inverse gamma prior distributions were used for all variance parameters, while all regression parameters were assigned Gaussian prior distributions with a mean of zero. For the country-level predictor regression coefficients, we utilized a regularization prior distribution similar to ridge regression by setting the prior variance of the Gaussian distribution equal to a shared and unknown variance parameter. This allowed us to deal with the large number of control variables under consideration and potential correlation between them. The remaining regression parameters (i.e., intercept and slope for vaccine effectiveness as a function of efficacy) were assigned a weakly informative distribution by specifying a prior standard deviation of 100 for the Gaussian distribution. All analyses were performed in R (Vienna, Austria). Code is available at https://github.com/vepitzer/rotaVEprediction.

### Model validation

We performed extensive model validation to assess the robustness of our results. First, we examined whether the inclusion of the covariates significantly improved the predictive ability of the model by comparing the performance of the full model (with all predictors and random effects) to a reduced version of the model with random effects but no predictors. Secondly, we performed a modified leave-one-country-out validation to fairly validate the predictions and investigate the model’s performance when used to predict in countries that contribute no data. We systematically removed all studies from an entire country from the dataset, fitted the model using the remaining studies, and then used the fitted model to predict vaccine efficacy and effectiveness for the studies from the excluded country. This closely resembles what we aim for in practical applications of this newly developed model (i.e., accurate and robust predictions for countries with no data). Finally, we conducted a traditional 5-fold leave 20% out cross-validation, randomly sampling 20% of effect estimates to leave out and using the remaining 80% to predict the withheld sample. Model performance was assessed by examining the correlation between the observed and predicted vaccine efficacy and effectiveness estimates and by calculating the root mean square error (RMSE) between the observed and predicted values. To evaluate the robustness of the model in terms of correctly characterizing uncertainty, we also calculated the percent of the 95% prediction intervals that contained the excluded values (i.e., empirical coverage).

### Model predictions for vaccine efficacy and effectiveness

Predictions were generated from the posterior predictive distribution of vaccine efficacy and effectiveness given the most recent (2023) predictor values of the specific country. We compared the range of the predictor values in countries with and without observed vaccine efficacy and effectiveness estimates to make sure that we were not extrapolating beyond the range of the observed predictors used in model fitting. For countries belonging to a WHO region not included in the dataset, the corresponding regional random effect values were drawn from the random effect distribution specified in the modelling framework using the collected posterior samples, ensuring that uncertainty for these predictions were appropriately inflated. Overall, this process yielded 100,000 samples from the posterior predictive distribution, which we summarized using posterior means and standard deviations for each country. We further summarized predictions by WHO region, using the median of the posterior country means and computing the credible intervals using the highest density interval.

### Ethics

The study utilized publicly available data that did not include individually identifiable information and therefore does not constitute human subjects research.

### Role of the funding source

The funders had no role in study design; the collection, analysis, and interpretation of data; writing of the report; or the decision to submit the paper for publication.

## Results

**Fig. 1:**
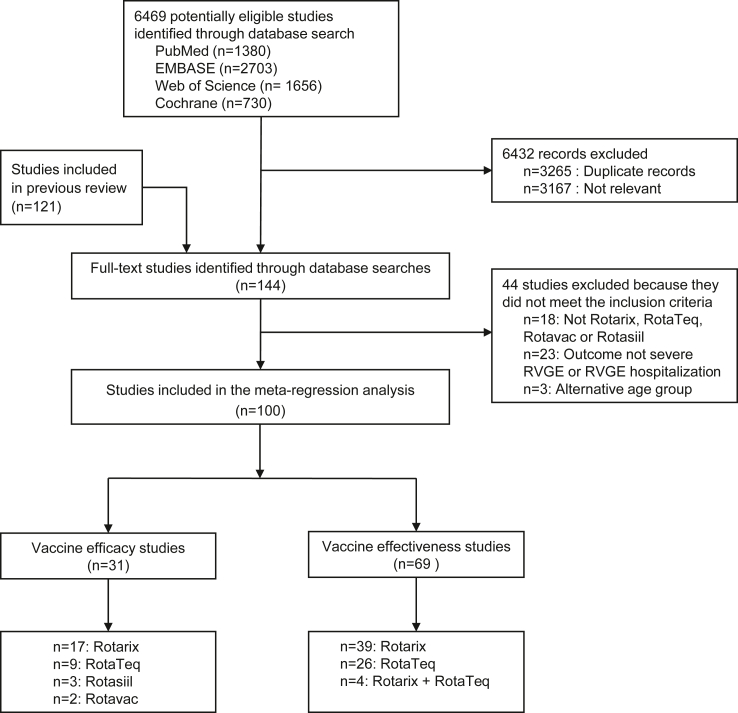
**Study selection process.** A previous systematic review identified 121 full-text articles,^20^ and 23 additional studies were identified for full-text review between July 1, 2020, and October 16, 2024. After the selection process, 31 randomized controlled trials, 66 case-control and 3 cohort studies were included. Inclusion and exclusion criteria are provided in Supplementary Table S1.

We identified a total of 144 full-text studies from the search strategy, out of which 100 met the inclusion criteria and were incorporated into our analysis (Supplementary Table S1). Fig. 1 shows the flow diagram of the study selection process. We identified nearly twice as many studies providing estimates for effectiveness (n = 69) compared with efficacy (n = 31). There exists a significant disparity in the study distribution across the available vaccines, with fewer studies available for the newer vaccines Rotasiil (n = 3) and Rotavac (n = 2) and more studies available for Rotarix (n = 56) and RotaTeq (n = 35), plus four studies that included both Rotarix and RotaTeq.

The included studies come from 56 countries, with a total of 164 observations; 62% (101/164) were country-specific observations, while the remaining 38% (63/164) were from multi-country studies. Almost half of the observations were from the Americas region (73/164 observations, 45%), with the fewest estimates in the South-East Asian region (7/164 observations, 4%) and only one observation from the Eastern Mediterranean region. Overall, 59% of the included studies were rated as high quality across all assessed criteria, 23% were of medium quality, and 18% were of low quality (see Supplementary Appendix 2).

There was wide variation in the study-specific estimates of rotavirus vaccine efficacy and effectiveness (Supplementary Appendix 2). Study-specific estimates of vaccine efficacy ranged from 17.6% in Mali (95% confidence interval (CI): −22.9%, 45.0%)^25^ to 100% in Japan (95% CI: 55.4%, 100%).^26^ Study-specific estimates of vaccine effectiveness ranged from 38% in Burkina Faso (95% CI: −165, 86%)^27^ to 98.4% in the United States (95% CI: 87%, 100%).^28^

### Model predictors, fitting, and validation

**Fig. 2:**
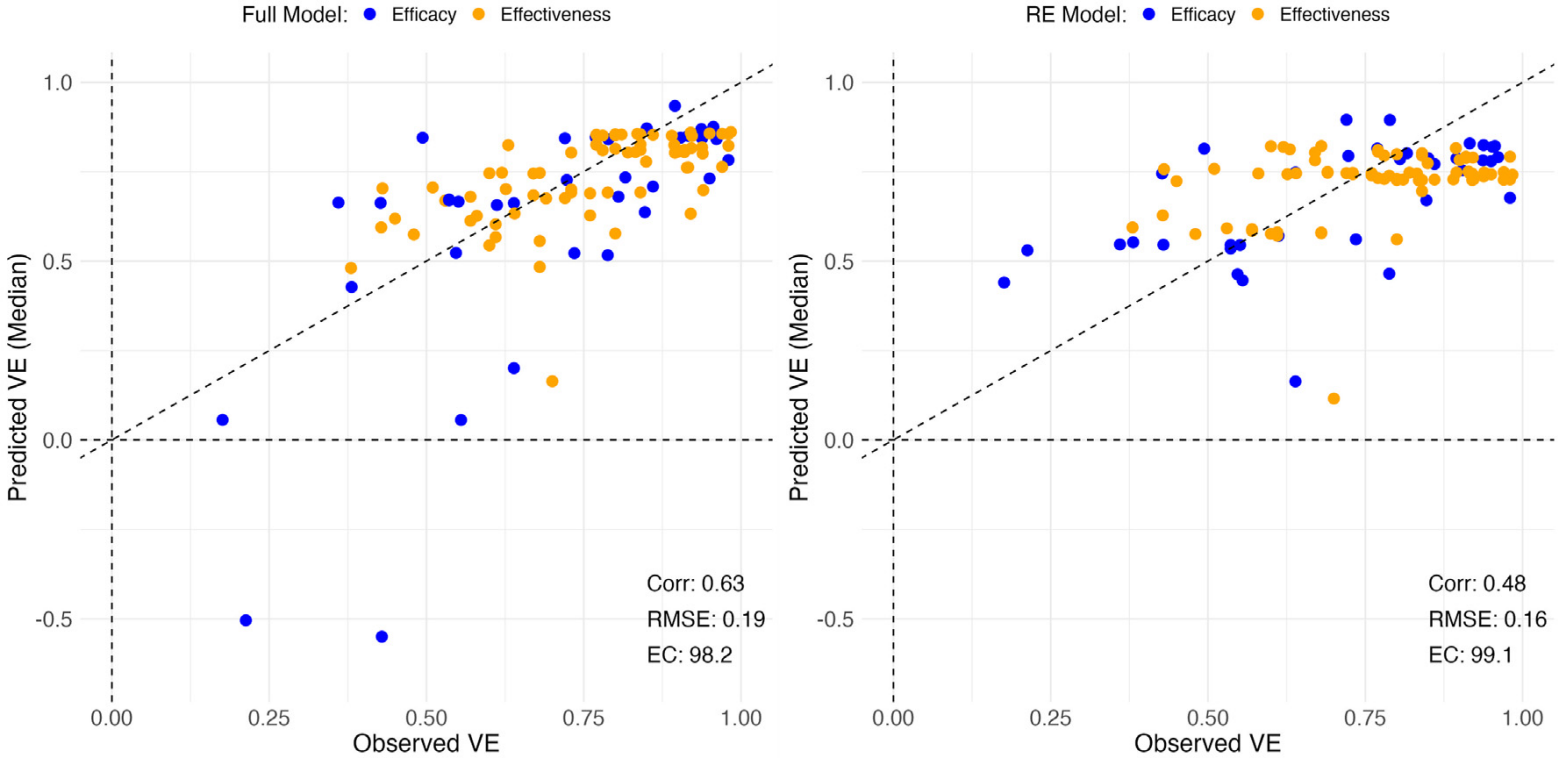
**Modified leave-one-country-out validation for the full and reduced models.** Left panel shows the performance from the full model which incorporates all predictors and World Health Organization (WHO) region-specific random effects, while the right panel shows the performance from the reduced model, with WHO region-specific random effects but no predictors. All studies from an entire country were systematically removed from the dataset, the model was fitted using the remaining studies, and then the fitted model was used to predict vaccine efficacy and effectiveness (VE) for the studies from the excluded country. The observed vaccine efficacy (blue) against severe rotavirus-associated gastroenteritis (RVGE) and vaccine effectiveness against hospitalized RVGE (orange) (1-RR, with RR the relative risk in vaccinated versus unvaccinated infants) for countries that were excluded from the fitted model is plotted on the x-axis, while the model-predicted vaccine efficacy or effectiveness for the excluded country is plotted on the y-axis. The dashed diagonal line indicates a perfect correspondence between observed and predictive values. The negative predicted VE values correspond to a study from Mali when this country is excluded from the model fitting. Abbreviations: Corr: correlation; RMSE: root mean squared error; EC: Empirical coverage.

Model predictors were highly correlated (Supplementary Fig. S2 in Supplementary Appendix 1), and none of the predictors were significantly associated with rotavirus vaccine efficacy in the full model (Table 1). However, the included predictors together with the random effects did improve predictive performance (Fig. 2 and Supplementary Table S2 in Supplementary Appendix 1). Performances from the modified leave-one-country-out validation demonstrated a high correlation between the observed and predicted vaccine efficacy and effectiveness values from the full model (r = 0.63, RMSE = 0.19), which was better than the performance of the model with no predictors and random effects only (r = 0.48, RMSE = 0.16) (Fig. 2 and Supplementary Table S2 in Supplementary Appendix 1). Additionally, the 95% prediction intervals included 98.2% of the observed vaccine efficacy and effectiveness estimates, suggesting that the intervals may be useful in quantifying uncertainty in out-of-sample predictions. The results from the 5-fold leave 20% out cross-validation were comparable (r = 0.71, RMSE = 0.13; Supplementary Fig. S3 in Supplementary Appendix 1). As a sensitivity analysis, we ran a univariate analysis with diarrhoea prevalence, GDP, and under five mortality as single predictors. We found that diarrhoea prevalence, GDP, and under five mortality were significantly associated with rotavirus vaccine efficacy in univariate analyses, though the predictive performance was lower for diarrhoea prevalence and under five mortality, and comparable for GDP when compared to the full model with all predictors (Supplementary Table S2 in Supplementary Appendix 1).

**Table 1:** Association between the predictor variables and the relative risk of severe rotavirus-associated gastroenteritis among vaccinated versus unvaccinated infants in the full model.

Predictor variable (Standard Deviation)	Relative risk^a^ (95% credible interval)
Diarrhoea prevalence among children <5 years of age (13.4)	1.09 (0.86, 1.33)
Population density (log transformed) (1.6)	0.98 (0.80, 1.17)
Gross domestic product per capita (log transformed) (6.1)	0.92 (0.56, 1.23)
Percent of the population using at least basic drinking water^b^ (20.4)	0.97 (0.79, 1.17)
Percent of the population using at least basic sanitation^c^ (29.8)	0.97 (0.74, 1.23)
Percent of the population living in extreme poverty (17.6)	1.00 (0.79, 1.21)
Oral polio vaccine coverage (38.8)	1.23 (0.93, 1.58)
Under 5 mortality rate (27.1)	1.22 (0.97, 1.49)
Antibiotic usage (7.2)	0.93 (0.70, 1.14)
Duration of study follow-up^d^ (categorized as ≤2 years or >2 years, with ≤2 years as the reference category)	1.07 (0.71, 1.47)

a The outcome measure is the relative risk of severe rotavirus-associated gastroenteritis in vaccinated versus unvaccinated infants, which is equal to 1 minus the vaccine efficacy. The interpretation is one standard deviation increase in covariate value leads to a β increase in the true log(RR).

b Defined as drinking water from an improved source, provided collection time is not more than 30 min for a roundtrip.

c Defined as use of improved facilities which are not shared with other households.

d For case-control studies, this variable was defined as age groups included in the study, with the reference group representing studies limited to cases and controls ≤2 years of age.

We assumed a linear relationship between the log-transformed relative risks and odds ratios, as outlined in our model description (Supplementary Appendix 1). Notably, our results suggest that there is less variability between countries in vaccine effectiveness than vaccine efficacy. When efficacy exceeded 60%, estimates of vaccine effectiveness tended to be lower than vaccine efficacy; conversely, when vaccine efficacy was low, effectiveness was predicted to be higher (Supplementary Fig. S4 in Supplementary Appendix 1). In a sensitivity analysis, we tested the assumption of linearity by assuming a quadratic relationship between the log-transformed relative risks and odds ratios and results were comparable (Supplementary Fig. S4 in Supplementary Appendix 1).

### Model predictions and geographic heterogeneity

**Fig. 3:**
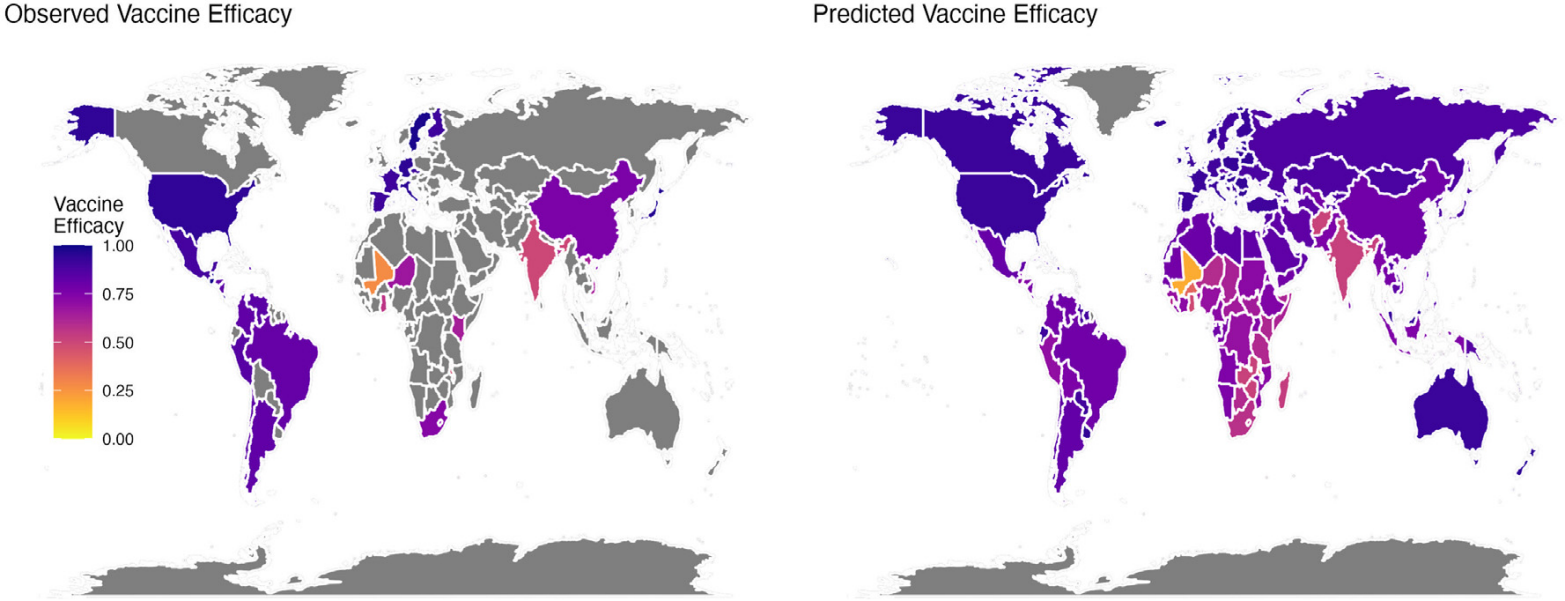
**Observed and model-predicted vaccine efficacy.** The left map shows the observed vaccine efficacy estimates against severe rotavirus-associated gastroenteritis from published randomized controlled trials, while the right map shows the model-predicted vaccine efficacy for 194 countries.

**Fig. 4:**
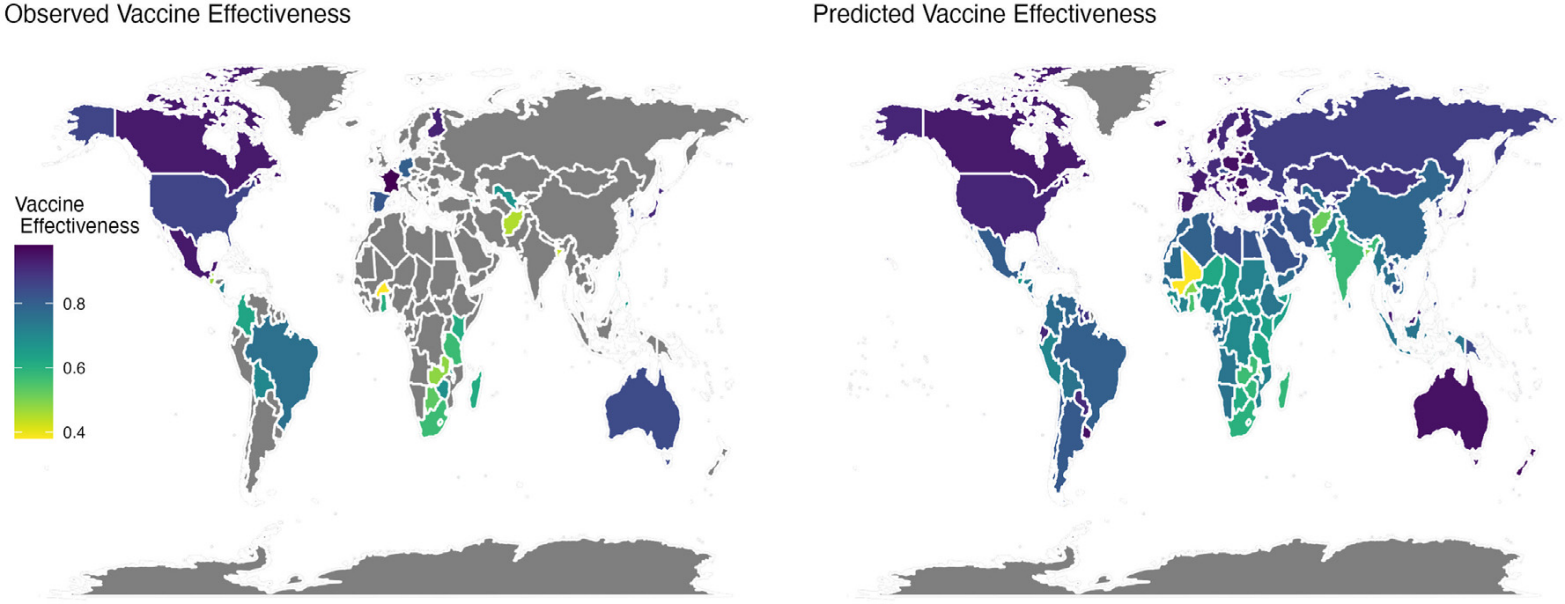
**Observed and model-predicted vaccine effectiveness.** The left map shows the observed vaccine effectiveness estimates against hospitalization with rotavirus-associated gastroenteritis from published case-control or cohort studies, while the right map shows the model-predicted vaccine effectiveness for 194 countries.

Across the 194 country-specific predictions of vaccine performance, vaccine efficacy ranged from 18.6% to 94.3% and vaccine effectiveness ranged from 42.7% to 85.7% (Fig. 3, Fig. 4 and Supplementary Appendix 3). The lowest vaccine efficacy and effectiveness was predicted for Mali (efficacy: 18.6%, 95% prediction interval (PI): −28.6%, 47.8%; effectiveness: 42.7%, 95% prediction interval (PI): −6.6%, 66.6%). The highest vaccine efficacy and effectiveness was predicted for Greece (efficacy: 94.3%, 95% PI: 81.7%, 98.5%; effectiveness: 85.7%, 95% PI: 71.5%, 93.3%). There was greater uncertainty associated with predictions of vaccine efficacy compared to vaccine effectiveness, as reflected in the wider 95% PIs.

**Fig. 5:**
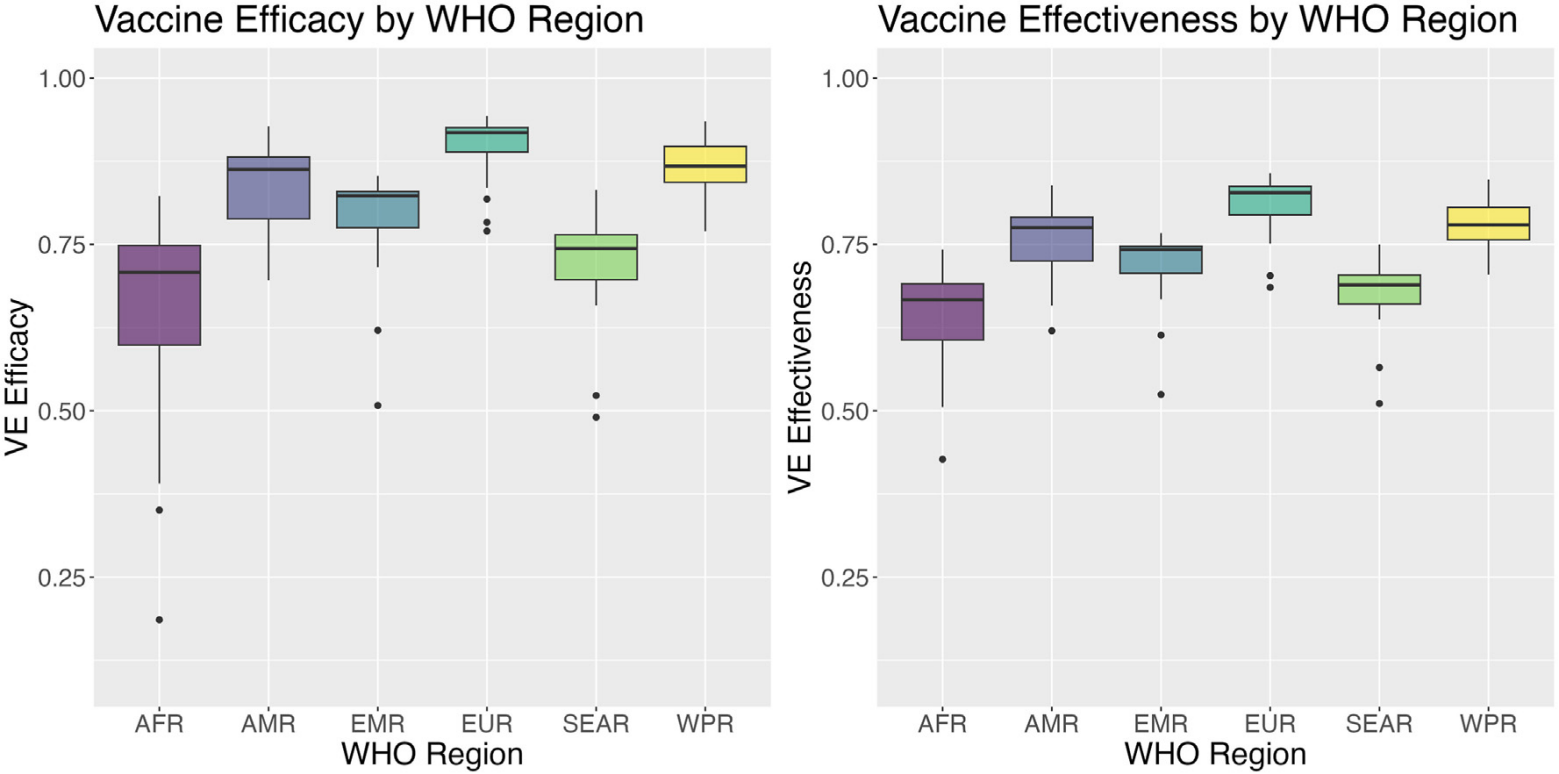
**Model-predicted vaccine efficacy and effectiveness by World Health Organization (WHO) region.** The left panel shows the box plots for the model-predicted vaccine efficacy estimates, while the right panel shows the box plots for the model-predicted vaccine effectiveness estimates by WHO region (African Region [AFR], Region of the Americas [AMR], South-East Asian Region [SEAR], European Region [EUR], Eastern Mediterranean Region [EMR], and Western Pacific Region [WPR]). The boxes represent the interquartile ranges, while the horizontal lines represent the median values within each region, and the vertical lines represent the 95% country-level median estimates; black dots represent the outlier predictions within each region.

Vaccine performance varied by WHO region (Fig. 5). Higher vaccine efficacy and effectiveness was predicted across the Americas, Europe, and Western Pacific regions (AMR efficacy median value: 82.8%, 95% PI: [72.5%, 88.7%]; AMR effectiveness: 75.0% [67.7%, 80.5%]; EUR efficacy: 89.6% [77.7%, 95.0%]; EUR effectiveness: 80.7% [72.0%, 86.6%]; WPR efficacy: 85.6% [72.3%, 92.2%]; WPR effectiveness: 77.2% [67.6%, 84.1%]). Meanwhile, decreased vaccine performance was predicted across the African and South-East Asian regions (AFR efficacy: 65.0% [28.4%, 79.9%], AFR effectiveness: 63.7% [44.4%, 75.1%]; SEAR efficacy: 70.1% [29.7%, 85.9%], SEAR effectiveness: 66.0% [44.8%, 78.6%]). Greater uncertainty was also associated with the African, Southeast Asian, and Eastern Mediterranean regions (Supplementary Appendix 3). In particular, the Eastern Mediterranean region, for which there were no studies conducted on vaccine efficacy and only one observed estimate of vaccine effectiveness, exhibited the greatest range of predictions across countries as well as the most uncertainty in predictions (EMR efficacy: 78.1% [1.3%, 92.6%], EMR effectiveness: 71.5% [39.7%, 83.9%]).

## Discussion

Variability in the efficacy and effectiveness of rotavirus vaccines has been observed in countries across the world, with higher vaccine performance generally observed in high-income and low-child-mortality countries.^20^^,^^29^, ^30^, ^31^ To better quantify and predict the efficacy and effectiveness of rotavirus vaccines at the country level, we developed a comprehensive meta-regression framework incorporating widely available indicators of economic and social development. Furthermore, we incorporated uncertainty in our estimates, capturing variability at both the study and country level. The robustness of our framework was confirmed through rigorous validation tests, demonstrating its high predictive performance. Our analysis provides country-specific estimates for both vaccine efficacy and effectiveness, offering valuable evidence for decision-making processes concerning the introduction of rotavirus vaccines in countries yet to adopt them.

Our findings indicate substantial variations in both vaccine efficacy and effectiveness across countries and WHO regions. The variability in vaccine efficacy and effectiveness is generally consistent with the spatial patterns of the predictors, emphasizing their association with the performance of rotavirus vaccines. Specifically, the regions of AMR, EUR, and WPR exhibited higher predicted values, contrasting with lower values predicted for AFR and SEAR, as has been noted previously.^32^ Other meta-analyses have also described variability in rotavirus vaccine efficacy and effectiveness by income groups^20^ and child mortality levels.^6^^,^^29^^,^^30^ We build on these analyses by establishing a meta-regression framework that can be used to predict variability in rotavirus vaccine performance across all countries based on additional widely available predictors that are known or hypothesized to be associated with rotavirus vaccine effectiveness.^33^^,^^34^ Notably, studies have shown the interference of OPV with rotavirus efficacy.^14^^,^^35^ High rates of co-infection can also lead to lower estimates of rotavirus vaccine efficacy,^36^ and high microbiome diversity and low length-for-age z-score have been associated with lower immune responses to rotavirus vaccines.^37^^,^^38^ Within Ghana, the performance of the Rotarix vaccine was modest and the estimated vaccine response rate was lower in regions characterized by a high burden of diarrhoea and inadequate WASH infrastructure.^7^^,^^39^

Our model assumed a linear relationship between the log-transformed relative risks and odds ratios, utilizing the relative risk as a predictor for the odds ratio, which is important for translating between vaccine efficacy as measured under the idealized conditions of clinical trials and vaccine effectiveness measured in real-world settings. The latter measure may be considered more relevant to policymakers evaluating the potential impact of rotavirus vaccines in their respective regions or countries. Interestingly, we found that vaccine effectiveness was predicted to be lower than vaccine efficacy when the efficacy exceeded 60%, which is to be expected given the broader population targeted and potential for suboptimal delivery when vaccines are used in practice.^40^ However, vaccine effectiveness was predicted to be higher at lower values of vaccine efficacy. Both vaccine efficacy and effectiveness may be underestimated in high-transmission settings due to the build-up of immunity from natural infection among unvaccinated individuals, which may be partly mitigated by the impact of vaccination on reducing transmission when rolled out on a population level.^41^^,^^42^ Our approach has the potential to be extended to predict the effectiveness of next-generation rotavirus vaccines from clinical trial data and can also be applied to other vaccine-preventable diseases, provided that the potential predictors of vaccine efficacy are known.

Our framework provides country-specific estimates for both vaccine efficacy and effectiveness, which is a valuable resource for decision-makers contemplating the introduction of rotavirus vaccines in countries burdened by rotavirus but lacking a clear vaccination strategy and context-specific data.^43^ Nevertheless, it is crucial to acknowledge the substantial uncertainty surrounding our predictions, stemming from limited vaccine efficacy data and sparse data on predictor variables. This resulted in larger prediction intervals for countries in AFR and EMR. To mitigate this uncertainty, our framework could be combined with expert opinions from local advisory groups and enhanced with higher-quality data for the predictor variables.

It is important to note a few limitations to our analysis. To ensure prediction accuracy, we selected predictor variables known or hypothesized to be associated with rotavirus vaccine performance, including socioeconomic and demographic indicators like GDP, under-five mortality, population density, poverty, and WASH indicators. Additionally, we included OPV coverage, considering evidence of its potential interference with rotavirus vaccine response.^14^^,^^35^ Notably, we were unable to identify specific predictors that could independently explain the observed heterogeneity. The lack of significance of the individual predictors may in part be explained by the fact that we are attempting to attribute subject-level variability in the response to vaccination to country-level predictors, resulting in ecological bias. However, decision-making on vaccine policy is typically done at the country level, and when combined and coupled with WHO region random effects, these predictors significantly contributed to the predictive power of our model, as demonstrated by our modified leave-one-country-out validation. Additionally, the directions of the estimated associations were generally consistent with the hypothesized relationships, e.g., higher OPV coverage was associated with higher relative risk and thus lower vaccine efficacy. Furthermore, we found that diarrhoea prevalence, GDP, and under-five mortality exhibited statistical significance in univariate analyses. If our goal was to identify specific predictor associations with vaccine performance, highly collinear predictors would need to be excluded. When considered collectively, these predictors effectively explained the heterogeneity in vaccine performance and contributed to the predictive power of our model. Also, we selected predictor variables at the national level, despite the high heterogeneity observed sub-nationally, and this could explain the heterogeneity observed in our estimates. We did not include the rotavirus genotype distribution as a potential predictor in our model despite potential differences in vaccine efficacy against certain genotypes because data on the genotype distribution are not available for all countries, and genotype distributions change over time and may be affected by vaccine introduction.^44^^,^^45^ Finally, we assumed vaccine performance did not vary across the four available vaccines because there were relatively few estimates of efficacy and effectiveness for Rotavac and Rotasiil (and none from high-income countries) and fewer estimates of vaccine effectiveness for RotaTeq from LMICs.

In conclusion, our study presents a robust tool for estimating country-specific vaccine efficacy and effectiveness, offering valuable guidance for decision-making processes at the country level when complemented with expert opinions from local advisory groups. This framework can be readily applied to other vaccines and settings characterized by geographical heterogeneity in vaccine performance. However, for enhanced representation, particularly in LMICs, additional data from clinical trials and vaccine effectiveness studies should be prioritized.

## Contributors

Conceptualization: OP, EOA, ES, VEP. Literature search: YL, ZP. Methodology: DMW, JLW. Data curation: YL, ZP. Data analysis: OP, EOA, ES, YL. Data interpretation: OP, EOA, ES, GEA, NAC, MIG, BAL, VEP. Funding acquisition: DMW, GEA, NAC, MIG, BAL, VEP. Supervision: DMW, JLW, VEP. Writing (original draft): OP, EOA, ES. Writing (reviewing & editing): all authors. OP, EOA, and VEP verified the underlying data. All authors read and approved the final version of the manuscript.

## Data sharing statement

All of the data and code necessary to reproduce this study are available in the Supplementary files and https://github.com/vepitzer/rotaVEprediction.

## Editor note

The Lancet Group takes a neutral position with respect to territorial claims in published maps and institutional affiliations.

## Declaration of interests

VEP was previously a member of the WHO Immunization and Vaccine related Implementation Research Advisory Committee. NAC is a National Institute for Health and Care Research (NIHR) Senior Investigator (NIHR203756). NAC is affiliated to the NIHR Global Health Research Group on Gastrointestinal Infections at the University of Liverpool; and to the NIHR Health Protection Research Unit in Gastrointestinal Infections at the University of Liverpool, a partnership with the UK Health Security Agency in collaboration with the University of Warwick. The views expressed are those of the author(s) and not necessarily those of the NIHR, the Department of Health and Social Care, the UK government or the UK Health Security Agency. DMW has received consulting and speaking fees from Pfizer, Merck, and GSK/Affinivax for work unrelated to this project; and DMW has received grants from Pfizer and Merck for work unrelated to this project. JLW has received consulting fees from Pfizer for work unrelated to this project. MIG is currently an employee of GSK; the content and views expressed are solely the responsibility of the author and not necessarily those of GSK. BAL reports personal fees outside the submitted work from Epidemiologic Research and Methods, LLC; Hillevax, Inc; and Merck Sharpe & Dohme. The content is solely the responsibility of the authors and does not necessarily represent the official views of the National Institutes of Health.
