# Supplementary material for: Global estimates of rotavirus vaccine efficacy and effectiveness: a rapid review and meta-regression analysis
Source: eClinicalMedicine. 2025 Mar 4;81:103122. doi: 10.1016/j.eclinm.2025.103122 (PMC11925534; doi:10.1016/j.eclinm.2025.103122)

**Supplementary Appendix 1**

**Global estimates of rotavirus vaccine efficacy and effectiveness: a rapid review and meta-regression analysis**

Ottavia Prunas, PhD^1-2^*, Ernest O. Asare, PhD^3-4^*, Elizabeth Sajewski, PhD^3-4^*, Yueqi Li, MPH^3^, Zeaan Pithawala, MS^3-4^, Daniel M. Weinberger, PhD^3-4^, Joshua L. Warren, PhD^4-5^, George E. Armah, PhD^6^, Nigel A. Cunliffe, PhD^7^, Miren Iturriza-Gómara, PhD^7^, Benjamin A. Lopman, PhD^8^, Virginia E. Pitzer, ScD^3-4^^

**contributed equally*

*^*Corresponding author: Virginia Pitzer, [virginia.pitzer@yale.edu](mailto:virginia.pitzer@yale.edu), Yale School of Public Health, Yale University, New Haven, CT, USA.

##### ^1^ Swiss Tropical and Public Health Institute, Basel, Switzerland

##### ^2^ University of Basel, Basel, Switzerland

^3^ Department of Epidemiology of Microbial Disease, Yale School of Public Health, Yale University, New Haven, CT, USA

^4^ Public Health Modeling Unit, Yale School of Public Health, Yale University, New Haven, CT, USA

^5^ Department of Biostatistics, Yale School of Public Health, Yale University, New Haven, CT, USA

^6^ Noguchi Memorial Institute for Medical Research, University of Ghana, Accra, Ghana

^7^ Department of Clinical Infection, Microbiology and Immunology, Institute of Infection, Veterinary and Ecological Sciences, University of Liverpool, Liverpool, UK

^8^ Department of Epidemiology, Rollins School of Public Health, Emory University, Atlanta, GA

**Supplementary material**

1. **Model description**

**Model for observed study-specific estimates of vaccine efficacy and vaccine effectiveness:**

$${\ln\left( 1-{\hat{\mathrm{VE}}}_{z_{i},i} \right) = \hat{\theta}}_{i}|\theta_{i}\sim N\left( \theta_{i},{\hat{\sigma}_{i}}^{2} \right), i=1,\ldots,n;$$

- ${\hat{\mathrm{VE}}}_{z_{i},i}$: Vaccine efficacy (for $z_{i}=0$) or vaccine effectiveness (for $z_{i}=1$) estimate from study *i* (*n* total studies)
- $\hat{\theta}_{i}, {\hat{\sigma}_{i}}^{2}$: Log relative risk estimate and variance, respectively, of severe (hospitalized) rotavirus-associated gastroenteritis (RVGE) in vaccinated versus unvaccinated individuals from study *i* (for $z_{i}=0$); outcomes may be log odds ratios for the prevalence of vaccination in cases versus controls for vaccine effectiveness studies (for $z_{i}=1$)
- $\theta_{i}$: True value (unobserved latent process)

**Model for latent study-specific estimates of vaccine efficacy and vaccine effectiveness:**

$$\theta_{i}|\eta_{z_{i}1},\ldots,\eta_{z_{i}n_{c}},\sigma_{\theta z_{i}}^{2}\sim N\left( \sum_{j=1}^{n_{c}} w_{ij}\eta_{z_{i}j},\sigma_{\theta z_{i}}^{2} \right), i=1,\ldots n;$$

- $\eta_{z_{i}j}$: True log relative risk ($\eta_{0j}$) or log odds ratio ($\eta_{1j}$) for country *j* (unobserved latent process); $n_{c}$ total countries
- $w_{ij}$: The proportion of total people in study *i* that are from country *j*
- $\sum_{j=1}^{n_{c}} w_{ij}\eta_{z_{i}j}$: Weighted average of country-specific log relative risks (or log odds ratios)
- $\sigma_{{\theta z}_{i}}^{2}$: Describes study-level variability (e.g., two studies with the same weights can still have different true $\theta_{i}$ values due to differences in study design)

**Model for latent country-level log relative risks (1-vaccine efficacy):**

$$\eta_{0j}|\mu,\boldsymbol{\beta},\phi_{s\left( j \right)},\sigma_{\eta_{0}}^{2}\sim N\left( \mu+\mathbf{x}_{j}^{T}\boldsymbol{\beta}+\phi_{s\left( j \right)},\sigma_{\eta_{0}}^{2} \right), j=1,\ldots,n_{c};$$

- $\mathbf{x}_{j}$: Vector of country *j*-specific predictors (excludes intercept)
- $\phi_{s\left( j \right)}$: Random effect, where $s\left( j \right)$ is the super region that contains country *j* ($n_{s}$ total super regions)
  - $\phi_{k}|\sigma_{\phi}^{2}\sim N\left( 0, \sigma_{\phi}^{2} \right), k=1,\ldots,n_{s}$
- $\sigma_{\eta_{0}}^{2}$: Describes the country-level variability (e.g., two countries may have the same predictors but have different true $\eta_{0j}$ values because we do not account for every predictor that explains variability in this value)

**Model for country-level log relative risks (or log odds ratios) (1-vaccine effectiveness):**

$$\eta_{1j}|\gamma_{0},\gamma_{1},\eta_{0j},\sigma_{\eta_{1}}^{2}\sim N\left( \gamma_{0}+\gamma_{1}\eta_{0j},\sigma_{\eta_{1}}^{2} \right), j=1,\ldots,n_{c};$$

- $\gamma_{0}, \gamma_{1}$: Intercept and slope terms that connect outcomes from vaccine effectiveness studies in country *j* ($\eta_{1j}$) with outcomes from vaccine efficacy studies from country *j* ($\eta_{0j}$)
- $\sigma_{\eta_{1}}^{2}$: Describes country-level variability in this relationship (e.g., the relationship between outcomes from vaccine efficacy and vaccine effectiveness studies may not be perfectly described by this relationship)

**Prior distributions:**

- $\mu,\gamma_{0},\gamma_{1}\sim N\left( {0,100}^{2} \right)$
- $\beta_{j}|\sigma_{\beta}^{2}\sim N\left( 0,\sigma_{\beta}^{2} \right)$
- $\sigma_{\theta0}^{2}, \sigma_{\theta1}^{2}, \sigma_{\eta_{0}}^{2},\sigma_{\eta_{1}}^{2}, \sigma_{\phi}^{2},\sigma_{\beta}^{2}\sim Inverse Gamma\left( 0.01, 0.01 \right)$

1. **Rapid review**

We performed a rapid review to identify studies estimating rotavirus efficacy and effectiveness published until 16 October 2024 using four databases (PubMed, EMBASE, Cochrane and Web of Science). To do so, we updated a previous systematic review by Sun et al. [20], which ended on 1 July 2020, using similar search terms and inclusion/exclusion criteria (Table S1). We also cross-referenced our list of included studies against two other recent systematic reviews of vaccine efficacy [29] and vaccine effectiveness [30].

**Table S1.** The inclusion and exclusion criteria used for article selection.

| Criteria | Study population | Study design | Publication requirements | Outcomes |
| --- | --- | --- | --- | --- |
| Inclusion | Children up to 5 years of age    Completed the recommended vaccine schedule    Licensed vaccines: Rotarix (RV1), RotaTeq (RV5), Rotavac and Rotasiil    Followed recommended dosage and schedule for each vaccine | Randomized controlled trials  Cohort studies    Case-control studies  Studies covering at least one rotavirus season  Cases confirmed by the EIA or ELISA technique | Articles published up to 16 October 2024 | Vaccine efficacy against severe RVGE (Vesikari score >11)    Vaccine effectiveness against rotavirus- associated hospitalization    Information about the population of participants in the study |
| Exclusion | Studies using other rotavirus vaccines |  | Articles published after 16 October 2024 | Information provided did not allow for efficacy or effectiveness calculation |

***Search strategy***

We searched Embase, PubMed, the Cochrane Library, and Web of Science for studies including randomized clinical trials, cohort studies, and case-control studies that reported on vaccine efficacy and/or effectiveness. The search was conducted by two reviewers at two different time points; YL performed the initial search on October 1, 2022 for studies published since July 1, 2020; ZP performed an updated search on October 16, 2024 for studies published since October 1, 2022 using the following search terms:

PubMed query: (rotavirus) AND (vaccin*)

EMBASE query: rotavirus AND vaccin*

Web of Science query: (ALL=(rotavirus)) AND ALL=(vaccin*)

Cochrane query: rotavirus AND vaccin*

Any questions about whether a study met the inclusion/exclusion criteria were resolved by consultation with two additional reviewers (VEP and EOA). A total of 144 articles were considered for full-text review, with 100 articles included after meeting the study’s inclusion criteria (Figure 1 in the main text).

***Data extraction***

We developed a data extraction table for the included studies to capture all relevant study information necessary for the research (Supplementary Appendix 2). Information extracted included author, date, study design, study type, country, sample size, vaccine type, and outcome of interest (vaccine efficacy or effectiveness, including point estimate and 95% confidence interval). Two reviewers (YL and ZP) extracted the data, and an additional reviewer (EOA) assessed the quality of the included studies.

**Table S2.** Modified leave-one-country-out validation comparing different sets of predictors.

| **Predictors** | **Random Effects** | **Relative risk* (95% credible interval)** | **Correlation** | **Root mean square error (RMSE)** | **Empirical coverage (EC)** | **Average length of prediction intervals** |
| --- | --- | --- | --- | --- | --- | --- |
| All | Yes | -- | 0.63 | 0.19 | 98.2 | 2.42 |
| None | Yes | -- | 0.48 | 0.16 | 99.1 | 2.65 |
| None | No | -- | -0.10 | 0.20 | 98.2 | 2.78 |
| Diarrhoea prevalence among children <5 years of age | Yes | 1.48 (1.02,1.98) | 0.59 | 0.15 | 97.3 | 2.29 |
| Gross domestic product per capita | Yes | 0.12  (0.02,0.27) | 0.63 | 0.15 | 98.2 | 2.12 |
| Under 5 mortality rate | Yes | 1.56  (1.21,1.91) | 0.49 | 0.44 | 98.2 | 2.72 |

*The outcome measure is the relative risk (RR) of severe rotavirus-associated gastroenteritis in vaccinated versus unvaccinated infants, which is equal to 1 minus the vaccine efficacy. The interpretation is one standard deviation increase in covariate value leads to a $\beta$ increase in the true log(RR).

**Figure S1. A schematic flow chart of the model development.**


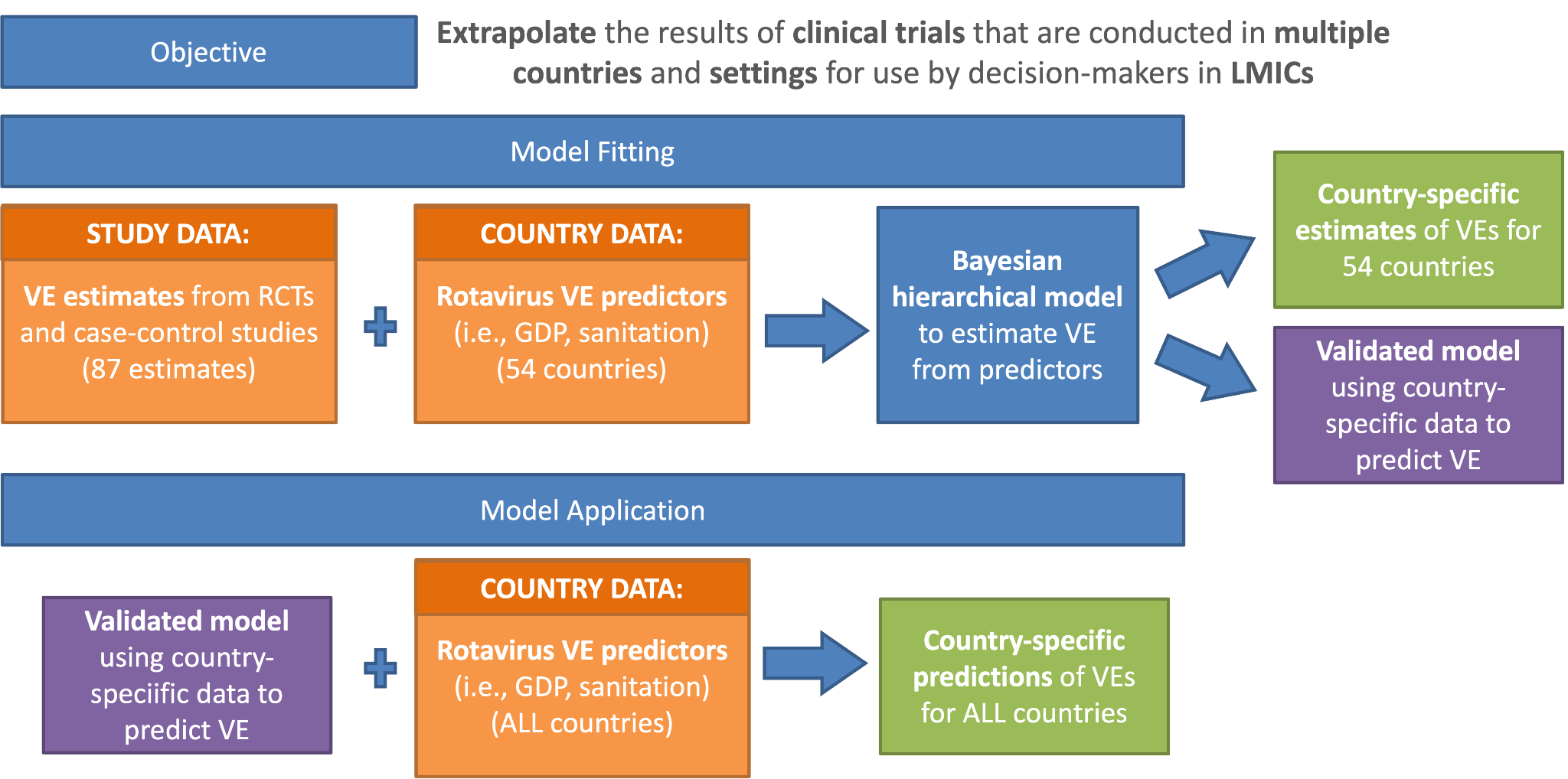


**Figure S2.** **Multi-collinearity of predictor variables**. The plot shows the correlation between the eight predictor variables included in the model, where size and colour saturation are indicative of the magnitude of correlation. The predictors are: pred1= diarrhoea prevalence among children <5 years old; pred2= log population density; pred3= gross domestic product (GDP) per capita; pred4= percent of the population using at least basic drinking water; pred5= percent of the population using at least basic sanitation; pred6= percent of the population living in extreme poverty; pred7= oral polio vaccine (OPV) coverage; pred8= vaccine efficacy/effectiveness follow-up in years; pred9= under-5 child mortality quintile; pred10=Antibiotic usage.


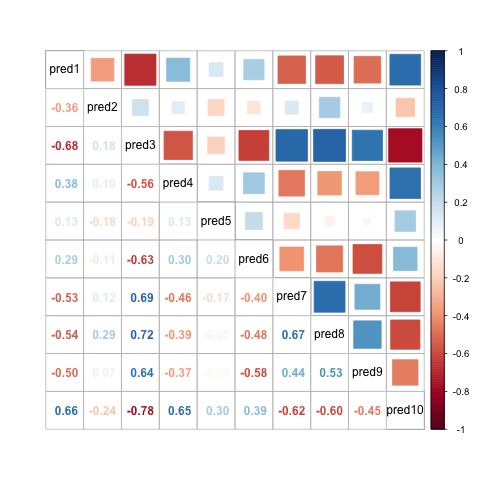


**Figure S3. 5-fold leave 20% out cross-validation.** Left panel shows the performance from the full model which incorporates all predictors and World Health Organization (WHO) region specific random effects, while the right panel shows the performance from the reduced model, with WHO region specific random effects but no predictors. Abbreviations: Corr: correlation; RMSE: root mean squared error; EC: Empirical coverage.

**Figure S4.** **Estimated relationship between vaccine efficacy and effectiveness**. Non-linear relationship between vaccine efficacy and effectiveness estimated from the model, where the vaccine effectiveness (log-scaled) is modelled as a linear function of vaccine efficacy (log-scaled) (left panel) and quadratic function (right panel). The dots represent modelled estimates of vaccine efficacy (VE1) and effectiveness (VE2) from the same country, coloured by the type(s) of data available in each country. The line represents the estimated regression trend line. For the linear assumption, the slope value is 1.72 (95% credible interval (CrI): 1.32, 2.23) and the intercept value is 0.66 (95% CrI: 0.39, 0.98); for the quadratic assumption, the slope value is 1.19 (95% CrI: 0.12, 3.19), the quadratic term is 0.81 (95% CrI: 0.42, 1.21) and the intercept value is 0.52 (95% CrI: 0.11, 1.08).


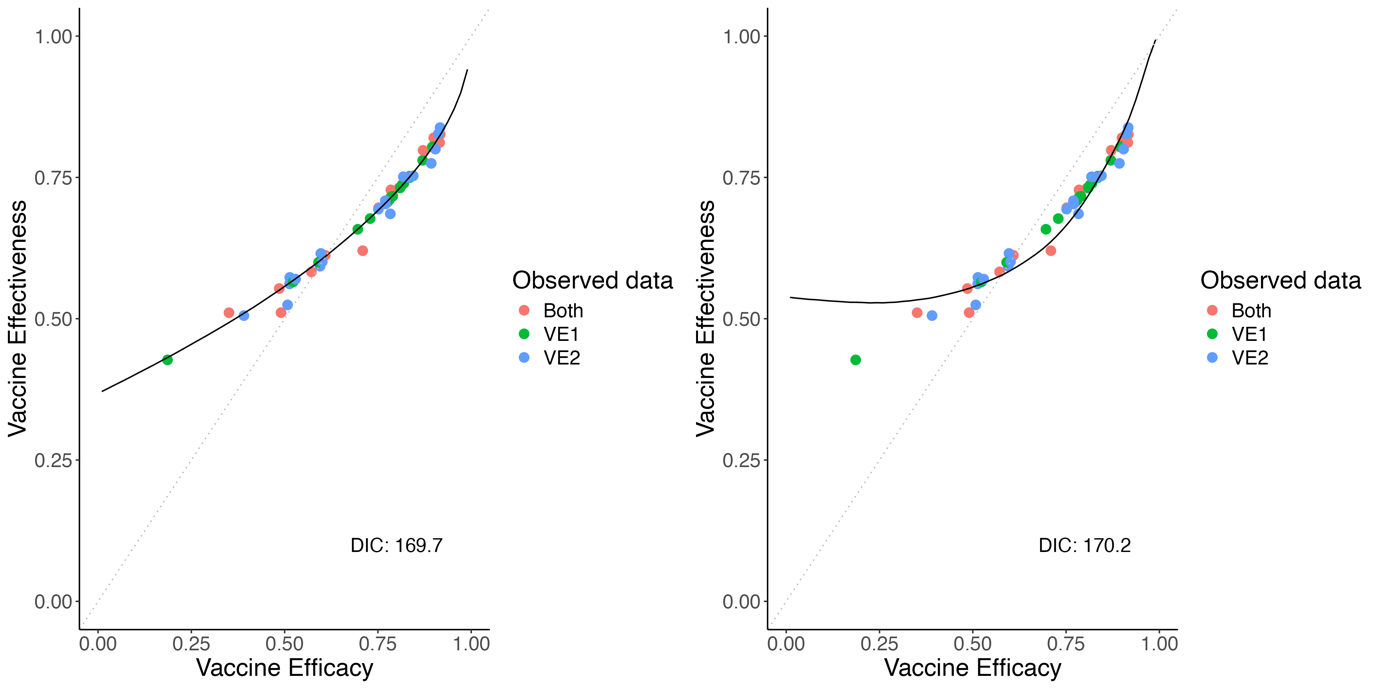

Supplement: Supplementary Appendix 1 [file mmc1.docx]
